# Paraffin for Fecal Impaction

**Published:** 1905-03

**Authors:** 


### PARAFFIN FOR FECAL IMPACTION.

Liquid paraffin has had a very limited sphere in therapeutics. It has frequently been used as a vehicle for menthol, camphor, thymol, and eucalyptol and occasionally creosote, terpinol and guaiacol. Further than this office it has been little known in medicine. For a number of years Dr. Theodore Potter has been making it a special study and recently read a paper before the Indianapolis Medical Society, lauding its virtues in the medical treatment of fecal impaction. Among the cases reported was one which gave every evidence of the necessity for a surgical operation, but was relieved by the use of liquid paraffin given internally. The bowels had not moved for three weeks, and liquid paraffin given in doses of three ounces each three hours with successful results. It is a solvent of fecal matter and is in no sense a cathartic, hence there is no griping when there is a movement of the bowels. It is neutral and not absorbable. Oil of vaseline and olive oil have frequently been used to accomplish the same results, but the argument set forth by Dr. Potter leads the writer to believe that this may prove a valuable contribution to therapeutics.—*Central States Medical Magazine.*

